# New Insights into the Application of 3D-Printing Technology in Hernia Repair

**DOI:** 10.3390/ma14227092

**Published:** 2021-11-22

**Authors:** Bárbara Pérez-Köhler, Selma Benito-Martínez, Verónica Gómez-Gil, Marta Rodríguez, Gemma Pascual, Juan Manuel Bellón

**Affiliations:** 1 Departamento de Medicina y Especialidades Médicas, Facultad de Medicina y Ciencias de la Salud, Universidad de Alcalá, 28805 Alcalá de Henares, Spain; barbara.perez@uah.es (B.P.-K.); selma.benito@uah.es (S.B.-M.); 2 Biomedical Networking Research Centre of Bioengineering, Biomaterials and Nanomedicine (CIBER-BBN), 28029 Madrid, Spain; veronica.gomezg@uah.es (V.G.-G.); marta.rodriguezma@uah.es (M.R.); juanm.bellon@uah.es (J.M.B.); 3 Ramón y Cajal Health Research Institute (IRYCIS), 28034 Madrid, Spain; 4 Departamento de Cirugía, Ciencias Médicas y Sociales, Facultad de Medicina y Ciencias de la Salud, Universidad de Alcalá, 28805 Alcalá de Henares, Spain; 5 Departamento de Ciencias Biomédicas, Facultad de Medicina y Ciencias de la Salud, Universidad de Alcalá, 28805 Alcalá de Henares, Spain

**Keywords:** additive manufacturing, bioactive mesh, hernia repair, mesh material, prosthesis, 3D-printing

## Abstract

Abdominal hernia repair using prosthetic materials is among the surgical interventions most widely performed worldwide. These materials, or meshes, are implanted to close the hernial defect, reinforcing the abdominal muscles and reestablishing mechanical functionality of the wall. Meshes for hernia repair are made of synthetic or biological materials exhibiting multiple shapes and configurations. Despite the myriad of devices currently marketed, the search for the ideal mesh continues as, thus far, no device offers optimal tissue repair and restored mechanical performance while minimizing postoperative complications. Additive manufacturing, or 3D-printing, has great potential for biomedical applications. Over the years, different biomaterials with advanced features have been successfully manufactured via 3D-printing for the repair of hard and soft tissues. This technological improvement is of high clinical relevance and paves the way to produce next-generation devices tailored to suit each individual patient. This review focuses on the state of the art and applications of 3D-printing technology for the manufacture of synthetic meshes. We highlight the latest approaches aimed at developing improved bioactive materials (e.g., optimizing antibacterial performance, drug release, or device opacity for contrast imaging). Challenges, limitations, and future perspectives are discussed, offering a comprehensive scenario for the applicability of 3D-printing in hernia repair.

## 1. Introduction

**Figure 1:**
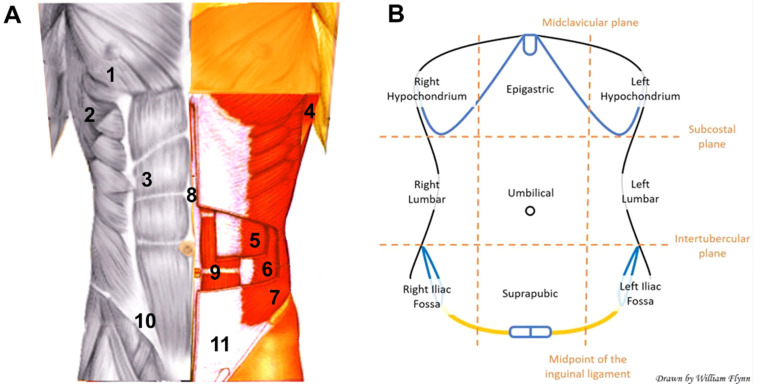
Anatomical structure of the anterior abdominal wall. (**A**) Principal muscles (1 pectoralis; 2 serratus anterior; 3 rectus abdominis; 4 latissimus dorsi; 5 transverse abdominis; 6 internal oblique; 7 external oblique) and other relevant components (8 linea alba; 9 tendinous intersection; 10 inguinal ligament; 11 external oblique aponeurosis). Original image by Scott Dulebohn [2] adapted with permission from StatPearls Publishing (Creative Commons Attribution 4.0 International License). (**B**) Diagram illustrating the different anatomical regions of the anterior abdominal wall. Original drawing by William Flynn [2] reproduced with permission from StatPearls Publishing (Creative Commons Attribution 4.0 International License).

The abdominal wall protects internal organs and stabilizes body posture while withstanding intraabdominal pressure variations caused by physiological events (e.g., breathing, digestion, coughing, urination, defecation), trunk movements, physical activity, or other efforts [1]. The abdominal wall (Figure 1) is basically comprised of layers of skeletal muscle tissue interwoven with connective fascias [2]. The outer side of the abdominal wall faces the subcutaneous tissue, while its inner surface is covered by peritoneum, which separates the wall muscles from the cavity organs [3,4]. All these components give rise to a functioning composite tissue structure of heterogeneous anatomical configuration [5]. The complexity of abdominal wall structure is important for its mechanical properties, which are influenced by many factors such as the (i) thickness of the different layers [6], (ii) presence of collagen and elastic fibers in connective tissues [7], (iii) anisotropy of the linea alba region [8], (iv) stiffness and strength of each individual component [9], (v) wall geometry [10], and (vi) non-linear response of soft tissues forming this composite-like structure [9].

One of the most frequent conditions that affects the integrity and functionality of the abdominal wall is herniation. A hernia arises when there is weakening, opening, or leakage of the abdominal muscles, causing loss of intraabdominal pressure and protrusion outward of the abdominal cavity contents [11]. In patients, these events may cause pain and reduced mobility among other incapacitating consequences. The clinical classification of hernias is complex and depends on several factors such as size, location, etiology, and variables influencing their development [12]. The genesis of a hernia is multifactorial involving genetic, biologic, or traumatic factors, or could be a postoperative complication of a laparotomy incision [13,14,15,16,17,18].

Surgical hernia repair can entail an open-surgery or laparoscopic approach. The high prevalence of hernias today determines that around 20 million patients undergo hernia repair annually worldwide [19,20]. The presence of comorbidities and risk factors (e.g., obesity, diabetes, hypertension, immunosuppression, previous surgeries, smoking) makes patients more likely to develop an abdominal hernia. According to a recent report, the Global Hernia Repair Devices and Consumables Market amounted to USD 4742 MM in 2019, and it is projected that this figure will increase to USD 6350 MM in 2027 [21]. 

Currently, the standard method for surgical hernia repair is the implant of a prosthetic material in the abdominal wall to close the defect, strengthen the abdominal muscles, and reestablish mechanical functionality [22]. Some implanted devices also reduce abdominal muscle stiffness, tissue ischemia, and even reherniation [11,23].

## 2. General Features of Prosthetic Materials for Hernia Repair

Over the years, intense research has been devoted to developing the ideal biomaterial for the repair of every hernia type [24,25]. Broadly speaking, the ideal material must be chemically inert, non-carcinogenic, non-allergenic, sterilizable, and easily adaptable to each specific need [26,27]. Once implanted, the device should elicit a minimal inflammatory reaction, reduce adhesion formation (under intraperitoneal conditions), achieve adequate vascularization and tissue integration, and resist bacterial infection [5,28]. Lastly, if the biomaterial is partially or fully resorbable, its degradation speed must parallel the progression of tissue repair to ensure that mechanical properties are properly restored [29,30].

Prosthetic devices for hernia repair are manufactured in a wide range of shapes differing according to characteristics such as the chemical nature of their components (synthetic, biological materials), architecture (laminar, reticular, composite), porosity (macroporous, microporous, non-porous), pore size (large pore, medium pore, small pore), weight g/m^2^ (lightweight, mediumweight, heavyweight), filament interweave (knitted, woven), number of filaments (monofilament, multifilament), and degradability (fully resorbable, partially resorbable, non-resorbable), among others [22,31].

## 3. Current Trends for Synthetic Meshes

There is currently a wide range of options available for the development of mesh materials for hernia repair applications. Within this spectrum, synthetic meshes are the main candidates outstripping collagen-based biomeshes [32], whose state of the art is not addressed here.

Synthetic meshes are usually the best option for abdominal wall-defect repair based on their adequate tensile strength and elasticity, susceptibility to tissue ingrowth and relatively good cost-effectiveness [33,34]. These devices can be elaborated using both non-resorbable or resorbable materials. Non-resorbable meshes are numerous and much used in hernia repair applications. These are stable devices that remain indefinitely in the body. The majority of these meshes are made of polypropylene (PP) [35], followed by polyester (PE) [36], and to a lesser extent, polytetrafluoroethylene (PTFE) [37], polyvinylidene fluoride (PVDF) [38], or polyurethane (PU) [39]. Resorbable meshes are less frequently used. These devices are made of one or more degradable materials which biodeteriorate in the short-term (days–weeks), mid-term (weeks–months) or long-term (months–years). Resorbable meshes are mainly composed of poly-4-hydroxybutyrate (P4HB) [40], polyglactin (PGL) [41], polylactic acid (PLA) [42], polyglycolic acid (PGA) [43], trimethylene carbonate (TMC) [44], polycaprolactone (PCL) [45], polyvinyl alcohol (PVA) [46], and combinations of these. 

The device mostly used for surgical hernia repair is macroporous reticular PP mesh. It was in the mid-20th century when Usher et al. started implanting PP devices in patients [47,48]. Since then, this has become the gold standard and is considered one of the best choices for repairing tissue–tissue defects [49,50,51]. However, despite good outcomes, PP implants can give rise to postsurgical complications such as wound dehiscence and migration [52], erosion [53], chronic pain [54], seroma formation [55], adhesions [56], bowel obstruction [57], fistula [58], or infection [59].

To minimize these complications, intense efforts have been made to study, design, and develop new prosthetic devices, and this has resulted in a broad range of meshes that have been patented, transferred to industry, or even marketed for clinical use [60]. This is because, despite technological and clinical advances, thus far it has not been possible to manufacture a single biomaterial with the precise characteristics needed to ensure optimal body–mesh matching and enhanced tissue repair while avoiding postsurgical complications or hernia recurrence. When selecting the best device to use, surgeons must consider multiple variables such as hernia type and its location, the patient’s physiopathological condition, and the surgical procedure to be carried out [61]. Consequently, there is still much interest in developing advanced materials and meshes for hernia repair.

## 4. Overview of 3D-Printing Technology for Biomedical Applications

Three-dimensional printing, or additive manufacturing, comprises a set of next-generation technologies that enables the fabrication of a device via the layer-by-layer deposition of a material. In this process, a successive series of bidimensional images is modeled using computer-aided design (CAD) software and subsequently used to create a digital model which serves to generate a 3D object whose geometry and shape are accurate [62]. To develop a patient-specific device via additive manufacturing, the process usually comprises four consecutive steps: (i) image acquisition, (ii) image partition or segmentation, (iii) adjustment of a 3D-model, and (iv) printing of the 3D-model [63]. Images are usually acquired using computed tomography (CT) or magnetic resonance imaging (MRI) approaches, following precise and specific parameters. The high resolution and contrast of the images enable the recording of the different tissues and the intricate shapes of the scanned area [64,65], allowing the generation of a 3D-model that mimics the body anatomy. Based on this model, a device that specifically matches the patient’s injured or damaged region is designed and 3D-printed with the aim to restore the biological and biomechanical functionality once implanted.

The starting point for 3D-printing technology arose in 1986, when Charles W. Hull developed stereolithography, a pioneer technique on which are based many of the 3D-printing methods currently in use [66,67]. Since then, additive manufacturing technology has continued to develop, giving rise to a wide range of methods and techniques for 3D-printing, as well as potential applications in the fields of industry, aeronautics, pharmacy, and biomedicine [68]. The recent boost in the manufacturing of 3D-printed implantable surgical devices has been described as the third industrial revolution [69] as it allows the design of materials specifically adapted to the patient´s needs, providing affordable, effective, reliable products able to mimic the complex anatomical structure of the human body [70]. 

According to the American Society for Testing and Materials (ASTM) and International Standard Rule ISO-17296-2:2017, all current 3D-printing strategies are classified into seven procedures: (i) fused deposition model (FDM), also known as material extrusion, (ii) powder bed fusion, (iii) vat photopolymerization, (iv) material jetting, (v) binder jetting, (vi) sheet lamination, and (vii) directed energy deposition [71,72]. Technical specifications, potential biomedical applications and limitations have been comprehensively reviewed by others [69,73,74,75,76].

The choice of procedure depends on many factors, such as the type of device and its application, manufacturing materials, time of production, cost, availability of equipment, and technical expertise. In the field of biomedical applications, FDM is the family of techniques most widely used for the development of devices [71], powder bed fusion has applications in drug delivery systems [77], and other approaches such as sheet lamination or directed energy deposition are of limited use in this field [71].

Along with the general conditions required for every prosthetic material [5,26,28], devices developed via 3D-printing technology must show specific features related either to the manufacturing process itself or the physicochemical properties of the compounds used. First, it is crucial to ensure both the printability of the raw material and its suitability to the printing method chosen. The device must be elaborated out of biocompatible compounds and, in cases where resorbable compounds are used, toxic sub-products must not be generated upon their degradation. Finally, once manufactured, the mechanical properties and shape of the printed device must be optimized for the intended application, recapitulating the biologic, physiologic, and biomechanical performance of native tissue [78,79].

Consistent with these requirements, advances in additive manufacturing have enabled the development of innumerable 3D-printed models, anatomical phantoms, surgical aids, devices, and prostheses for biomedical and tissue engineering applications. Over the years, a wide variety of biomaterials and constructs have been fabricated using this technology for hard and soft tissues, including but not limited to bone, skin, cartilage, cardiovascular system, skeletal muscle, solid organs, and nerves [72,80,81,82].

While most devices manufactured are experimental concepts tested in vitro and/or in vivo, few have been cleared by the US Food and Drug Administration (FDA). To date, the FDA’s Centers for Devices and Radiological Health (CDRH), Drug Evaluation and Research (CDER), and Biologics Evaluation and Research (CBER) have reviewed, evaluated, and cleared the clinical use of nearly 100 class-II, class-III, and patient-specific devices (e.g., joint, hip, spine, knee, cranial, or dental implants) manufactured through 510(k) processes [68,74,83,84] and also approved the fabrication of the first drug via additive printing [85]. Likewise, the FDA is currently revising the accuracy of the hardware and software (e.g., technology, model fidelity, safety, effectiveness, reliability, and reproducibility) used in biomedical 3D-printing [71]. Further, the creation of the FDA’s Humanitarian Use Device Program seeks to facilitate the immediate use of 3D-printed devices in patients suffering from rare life-threatening diseases [74].

## 5. Innovative 3D-Printed Meshes for Tissue Engineering Applications

**Figure 2:**
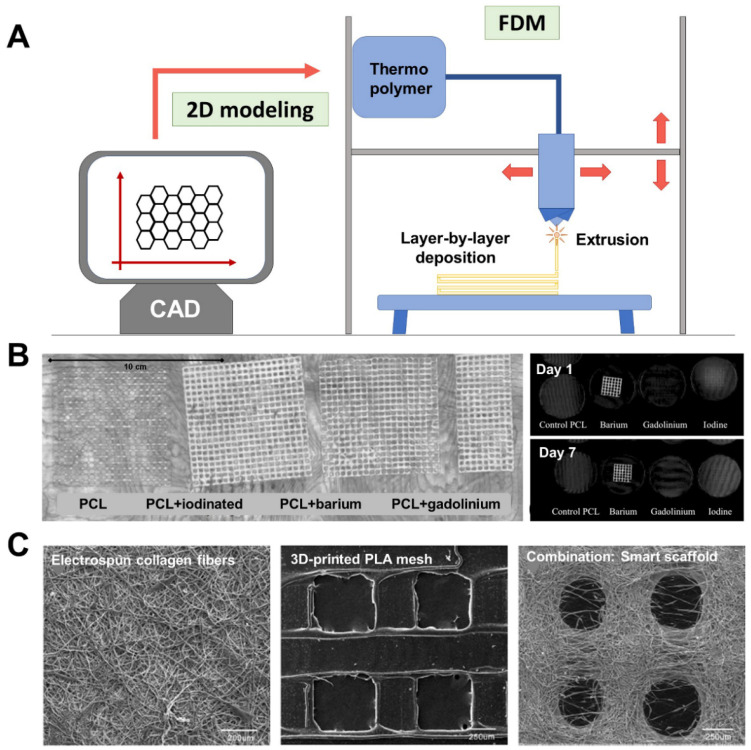
Illustrative examples of different devices for hernia repair fabricated by 3D-printing. (**A**) Schematic diagram of FDM additive-manufacturing technology. (**B**) Left panel: maximum-intensity coronal reconstruction of printed PCL meshes endowed with contrast agents (iodine-based, barium, gadolinium) for computed tomography (CT) traceability. Right panel: CT pictures showing coronal volume of the meshes after 1 and 7 days of culture on agar plates at 37 °C. Original pictures from [83] adapted with permission from BMC-Springer Nature (Creative Commons Attribution 4.0 International License). (**C**) Scanning electron microscopy (SEM) images of a smart scaffold elaborated by interleaving of a 3D-printed PLA mesh with electrospun type-I collagen fibers. Original pictures from [84] adapted with permission from BMC-Springer Nature (Creative Commons Attribution 4.0 International License).

As suggested by the latest literature reports (Table 1), the fabrication of hernia repair devices for biomedical and tissue engineering applications is currently in a stage of growth although the prototypes developed have not yet been cleared by the FDA. Polymer-extruding techniques such as FDM (Figure 2A) are the main methods being used to develop 3D-printed meshes. This technology is believed not to affect the chemical nature of the printed compounds, as they do not usually require post-curing stages [79]. With FDM, a given thermopolymer is heated to its melting point and subsequently extruded through a nozzle onto a platform in a layer-by-layer manner. Once created, each layer cools down, turns solid, and then a new layer of melted material is deposited on top of the previous one, faithfully reproducing the shape of the digitalized model [71].

Using this technology, different meshes and scaffolds with potential clinical application have been manufactured over the past few years. To aid their postimplant visualization in CT and MRI techniques, Ballard et al. developed PCL meshes containing iodine-based, gadolinium, or barium contrast agents [86] (Figure 2B). The idea behind this strategy was to apply the layer-by-layer approach to avoid the toxicity inherent to these compounds. By depositing contrast agents in the deepest areas of the mesh filaments, their diffusion to surrounding tissues would be minimized, reducing systemic dissemination of these chemicals. Results from this preliminary in vitro study revealed the good visibility of these meshes in CT scans, although not all the contrast agents tested showed the same traceability over time.

Additive manufacturing is currently being used in combination with other technologies to transform 3D-printed meshes into innovative scaffolds for tissue engineering applications. Recently, Yang et al. complemented FDM with electrospinning methods to develop a smart scaffold composed of alternating layers of printed PCL filaments and electrospun type-I collagen fibers [87] (Figure 2C). Scaffolds (3 × 2 cm) were assessed in vitro and in a full-thickness abdominal wall-defect model in the rat at 2, 4, and 12 postoperative weeks. Findings indicated that the added collagen stimulated migration of fibroblasts and tissue ingrowth into the scaffold, providing improved tissue repair in comparison with either bare PCL-printed meshes or commercial collagen-based biomeshes. Likewise, the scaffolds showed an improved biomechanical response once implanted in the abdominal wall. This pioneer approach is also appealing for tissue engineering applications other than hernia repair. Such is the case of a scaffold made of a printed PLA mesh covered with gelatin–PCL electrospun fibers recently developed for use as a mesh-like membrane covering for calvarial bone grafts. Outcomes in terms of mechanical strength and tissue incorporation have been promising [88]. 

The latest breakthrough in devices for tissue engineering of hernia defects consists of scaffolds able to modulate the inflammatory response that follows hernia repair. Shin et al. manufactured inflammation-modulating PLA scaffolds crosslinked with phosphate with the physiological ability to trap inflammatory cytokines [89]. The main objective pursued was to mitigate the proinflammatory environment in the abdominal wall that usually stimulates adhesion formation in intraperitoneal mesh repairs. Scaffolds were elaborated with different degrees of crosslinks and meticulously assessed in vitro for mechanical properties, cytokine trapping, swelling capacity, degradation, and toxicity. Adhesion prevention was preclinically analyzed in a rat model of ventral hernia repair (2 × 2 cm), comparing the performance of the developed bioscaffolds with that of a marketed PP mesh and a composite scaffold combining marketed PP and printed PCL. The scaffold showed excellent biomechanical performance along with optimal prevention of adhesion formation at two and four postoperative weeks, correlating with efficient cytokine trapping.

**Table 1:** Summary of the experimental research conducted to date to develop hernia repair mesh materials and scaffolds via additive-manufacturing technology. (Abbreviations: CT: computed tomography; FDM: fused deposition model; NP: not provided; PCL: polycaprolactone; PLA: polylactic acid; PP: polypropylene; PVA: polyvinyl alcohol; SEM: scanning electron microscopy).

Device Developed	FDM Printing Parameters	Main Experimental Procedures	References
PLA meshes containing contrast agents: iodine-based, gadolinium, or barium (1:10 *w*/*w*).	Extrusion temperature: 125–130 °C Speed: 7 mm/s Layer height: 0.2 mm Mesh pore size: NP	In vitro: CT imaging (120 kVp 120, 220 mAs). Stability of radio-opacity at body temperature: agar plate incubation (7 days, 37 °C).	[86]
Composite scaffolds made of printed PCL meshes and electrospun type-I collagen fibers.	Extrusion temperature: 190 °C Speed: 300 mm/min Layer height: 225 µm Mesh pore size: 800 µm Additional technology: electrospinning of type-I collagen (filament diameter 0.9 mm; flow 0.39 mL/min) alternating with PCL layers	In vitro: SEM visualization. Cytocompatibility (rat skin fibroblasts): proliferation studies (days 1, 3, 5, and 7 post-seeding). In vivo: Full-thickness abdominal wall defect repaired with scaffolds (2 × 3 cm) in male Sprague–Dawley rats (n = 54, three study groups). Clean surgery. Groups (3): PCL/PCL + electrospun collagen/Marketed collagen-based biomesh. Macroscopic, biomechanical and histopathological assessment at postoperative weeks 2, 4, and 14.	[87]
Inflammation modulating bioscaffold made of phosphate-crosslinked PVA.	Extrusion temperature: NP Speed: 10 mm/s Layer height: NP Mesh pore size: NP	In vitro: Mechanical testing. Crosslink reaction (3 phosphate concentrations: 15, 10, 7.5%). Swelling capacity over time (144 h). Material degradation. Cytocompatibility (human skin fibroblasts and microvascular endothelial cells): proliferation studies (72 h post-seeding). Trapping of proinflammatory cytokines (bead-based bioassay). In vivo: Ventral hernia defect (2 cm) repaired by the intraperitoneal implantation of scaffolds (2 × 2 cm) in male Sprague–Dawley rats (n = 36, three study groups). Additional implantation of bioscaffolds with modified surface charge (positive, neutral, negative; n = 6 each). Clean surgery. Groups (3): PLA bioscaffold/Marketed PP/Composite of marketed PP + PLA bioscaffold. Macroscopic, histopathological, and mRNA determination of cytokines at postoperative weeks 2 and 4.	[89]

## 6. 3D-Printed Bioactive Meshes for Hernia Repair

Additive manufacturing paves the way for the development of so-called bioactive biomaterials, which are cutting-edge prostheses with enhanced properties conferred by the presence of bioactive agents. The layer-by-layer fabrication system means that substances, compounds, drugs, and even living cells can be interleaved between layers of the printed material [90]. The printed layers and compounds used can be tailored to achieve a coordinated balance between drug release and device degradation [91,92], thereby enhancing tissue repair.

Most experimental research on these devices has sought to provide additional features that will confer 3D-printed meshes benefits over the devices currently available. For this purpose, the layer-by-layer deposition of material is a powerful tool for the development of bioactive meshes [93]. This approach pursues the local, controlled, sustained release of medication in the surgical area to optimize drug doses while minimizing systemic toxicity and other undesired side effects [74]. Depending on the final application, different drugs can be incorporated into these 3D-printed meshes such as antibiotics [94], immunosuppressants and chemotherapies [95], hormones [96,97], or growth factors [98] (Table 2).

The first proof of concept for 3D-printed bioactive meshes was provided by Weisman et al., who developed PLA filaments containing gentamicin or methotrexate as the starting point for the manufacture of synthetic biomedical implants with antibacterial or chemotherapeutic properties [94]. Later on, these authors printed gentamicin-loaded PLA meshes that were tested in vitro against *Staphylococcus aureus* and *Escherichia coli* [98]. Collectively, results revealed the feasibility of adding drugs to the manufactured device, and, importantly, that the FDM printing conditions (e.g., polymer melting temperature, extrusion procedure, printing time) did not affect drug activity. These promising findings set the stage for the additive manufacturing of bioactive meshes.

A recent development has been PCL meshes of two different pore sizes containing sodium alginate-encapsulated gentamicin [99]. The antibacterial activity of these devices was assessed in vitro against *E. coli*. In addition, a supra-muscular model of mesh implantation (2 × 2 cm) in the abdominal wall was established in female Wistar rats to assess host tissue incorporation in the printed meshes at seven days postimplant. The prototypes showed good antibacterial activity in vitro, as well as mild inflammation and early tissue repair in vivo. However, adhesions to these devices limit their intraperitoneal applicability.

Other antibiotics have been tested as candidates for their use in 3D-printed devices. Qamar et al. tested the use of ciprofloxacin to treat complicated abdominal or urinary tract infections [100]. These authors manufactured PP and PVA meshes of varying combinations of material composition, drug loaded, thread width, pore size, pore shape, and number of pores per surface unit (100 cm^2^). Thirty-two mesh configurations were assessed in vitro to determine mechanical strength and drug-release kinetics. The most adequate configuration was then preclinically tested in a rabbit model of incisional defect with the mesh implanted in contact with the abdominal cavity in a six-week study. Better performance was observed of the drug-loaded PP meshes in comparison with the resorbable PVA devices, particularly in terms of their mechanical properties, tissue incorporation, and inflammation. Interestingly, the addition of ciprofloxacin to printed PVA- but not PP meshes led to a reduction in tensile strength.

Similar meshes enhanced with bioactive properties have been additive manufactured to treat skeletal muscle conditions such as in pelvic floor reconstruction to prevent pelvic organ prolapse. In 2017, Tappa et al. developed smart PCL mesh-like scaffolds for the release of estrogen and progesterone, either alone or in combination [96]. Extruded meshes exerted optimal drug release profile, cytocompatibility, and bioactivity in vitro. More recently, Farmer et al. printed PU meshes with different pore shapes loaded with estradiol proangiogenic hormone to stimulate host-tissue vascularization, and tested the in vitro drug-release, physical, and mechanical properties of the devices in comparison with printed PP meshes [97]. The mechanical response was found to be influenced by both mesh chemical composition and geometry. Likewise, the presence of estradiol did not modify the elastic modulus of the printed PU meshes, which showed improved performance over the PP meshes.

## 7. Biomechanical Considerations for Developing 3D-Printed Meshes

As with other medical devices [102,103], additive manufacturing will help customize a biomaterial to create a patient-specific device. Theoretically, this approach allows tailoring of the chemical composition, morphology, pore size, thickness, and flexibility of the printed material, along with other key characteristics [22,74]. However, despite clear benefits, designing meshes by 3D-printing can be challenging for different reasons.

Among the special features characterizing the abdominal wall is that this is a dynamic, anisotropic structure constantly subjected to both changes in intraabdominal pressure and mechanical forces which are exerted in all directions [8,104,105]. To be compliant with the host tissue, implanted meshes must withstand abdominal pressure [106], maintain an appropriate balance between stability and stretchability [107], and exhibit adequate mechanical properties in terms of tensile strength, stiffness, and elasticity [22,24,108]. In effect, knitted patterns and pore geometry are core characteristics directly related to the biomechanical performance of the mesh [109].

Typically, meshes for hernia repair are elaborated using one or more knitted filaments which are interwoven to form a network of parallel and perpendicular strands [110]. Knitted meshes are likely to improve elasticity and provide anisotropy to the implant, which are important for implant stability and adapting the mesh response to the different horizontal, longitudinal, and transverse stress forces to which the abdomen is subjected [108]. Due to the layer-by-layer procedure of 3D-printing technology, printed meshes do not have genuine knits. This means a printed mesh will behave more like a woven than knitted fabric, making it more isotropic and less elastic than a knitted mesh [22]. This could thus compromise the compliance of a printed mesh.

Pore geometry and mesh weight are interconnected factors that play an important role in the biomechanical performance of a device. As mesh pores are irregular in shape, the variable pore size is given as the largest circular area contained within the pore, commonly known as effective pore size [22]. Usually, effective pore diameters greater than 1 mm are considered large, while those smaller than this threshold are small pores [11,24]. For mesh weight, although different classifications have been formulated, meshes weighing less than 50 g/m^2^ are generally described as lightweight, those of more than 80 g/m^2^ are heavyweight, and mediumweight meshes are somewhere within this range [24,111]. In most cases, meshes with large pores are lightweight and those with small pores are heavyweight, although not all marketed prostheses meet these criteria [112]. The consensus is that large-pored meshes enable better tissue ingrowth, cell migration, and vascularization, and give rise to a milder inflammatory response compared to those with small pores [24,33]. Likewise, lightweight meshes are more flexible and stimulate the formation of a looser, less fibrous connective tissue infiltrating the pores in comparison with heavyweight meshes, thus favoring compliance with host tissue [111]. Hence, the combination of large pores and light weight serves to minimize the amount of foreign body in the host tissue, enhancing mesh integration, modulating the inflammatory reaction, and providing adequate tensile strength to the implant [106,113]. Notwithstanding this, 3D-printing of lightweight meshes can be influenced by different factors, such as the total mass of thermopolymer extruded and the manufacturing method used. These parameters may modify the mechanical strength and elasticity of the printed device [114], leading to an impaired performance once implanted. For example, when the amount of polymer extruded is excessive, the printed mesh would display adequate pore configuration but a stiffer architecture than expected. Eventually, the host-tissue response of these meshes could increase the risk of forming tissue granulomas due to an inappropriate tissue ingrowth or even an exacerbated inflammatory response.

In the case of bioactive devices, the combination of thermopolymers and medications strives to improve some property of the printed mesh (e.g., antibacterial action, drug release, device opacity for contrast imaging). While this approach enables the optimization of drug concentration for an improved dosage, adding bioactive agents above a threshold level is likely to disrupt the physical properties of the printed polymer, thus modifying the device’s biomechanical properties [93]. Laboratory and preclinical studies are needed to assess the optimal combinations of these compounds required to create optimized safe devices for tissue engineering applications.

## 8. Future Perspectives for 3D-Printed Meshes

Additive manufacturing is driving the development of advanced, highly accurate, patient-specific implantable prostheses. This technology facilitates the design of complex devices that could not be fashioned using traditional approaches, and also allows for easy and quick surface modification of a preexisting device [102]. Although meshes for hernia repair have been constantly evolving, there are still some features that additive manufacturing could greatly improve upon.

Besides the development of next-generation bioactive meshes [74], 3D-printing could offer additional features to a mesh enhancing its compliance with host tissues. For instance, meshes are currently marketed provided with grips [115] or adhesives [116] for device self-fixation. This strategy avoids the host-tissue trauma caused by suture fixation and notably shortens the surgery time. With additive manufacturing, these structures could be easily incorporated in virtually all the meshes available, regardless of the device’s chemical composition or geometry. Another potential application of this technology could be focused on preventing adhesion formation when the mesh needs to adopt an intraperitoneal position. This complication can have severe consequences, especially when a synthetic reticular mesh is placed in contact with the intestinal loops [117]. Adding a printed layer of an antiadhesive compound could prevent adhesion formation. This strategy would be particularly useful to improve the behavior of the widely used reticular PP and PE meshes.

Besides synthetic thermopolymers, biological compounds such as cells, extracellular matrix proteins, and molecules can be used in additive manufacturing to create innovative devices and living biologically active tissue constructs [81]. This concept, known as bioprinting or biofabrication [95], has enormous potential for tissue engineering applications. Extruded biological hydrogels and bioinks provide an optimal environment for cell growth and migration and promote vascularization [81], altogether improving wound repair and tissue regeneration. Effectively, bioprinted skeletal muscle constructs have been successfully developed with promising outcomes [118] and could be candidates for the repair of abdominal wall defects.

The next step for additive manufacturing is so-called 4D-printing. This emerging approach seeks to resolve the limitations of 3D-printed devices to recapitulate the dynamics of living tissues by introducing “time” as a new factor [119]. In 4D-printing, smart thermopolymers capable of shape changes in response to physicochemical or biochemical stimuli (e.g., temperature, pressure, presence of molecules, pH) [68] can be extruded via FDM approaches. Stimuli-responsive polymers could be used to fabricate pioneer meshes with the ability to progressively adapt and respond to changes in the host-tissue environment, enhancing tissue ingrowth and implant compliance. Likewise, drug delivery systems could be optimized with this technology, enabling drug-loaded printed meshes to release their medication only and specifically when needed (e.g., release of antibiotics in the presence of bacterial toxins, release of cytokines and growth factors to stimulate cell migration and vascularization).

## 9. Conclusions

Additive manufacturing is an ongoing technology with enormous potential for biomedical and tissue engineering applications. In the hernia repair field, printed next-generation meshes are set to boost the development of accurate, patient-matching prostheses showing improved compliance with host tissue. The possibility to fine tune different features (e.g., chemical composition, shape, thickness, porosity, weight) will enhance the biomechanical performance of these meshes once implanted. Likewise, by incorporating additional properties (e.g., drugs, hormones, contrast agents) smart bioactive meshes will pursue reduction in some postoperative complications while improving host tissue incorporation and repair.

Strategies for 3D-printing of meshes are generating international databases and repositories for the sharing among clinicians and researchers of concepts, phantoms, models, or training devices. Encouraging results have been obtained thus far with these devices in both experimental and preclinical scenarios. More research is needed into the potential clinical applications of this appealing technology in hernia repair and to ensure the optimal biocompatibility, safety, and reliability of printed meshes for their FDA clearance and approval.

## Figures and Tables

**Table 2:** Summary of the experimental research conducted to date to develop drug-loaded bioactive hernia repair mesh materials via additive manufacturing technology. (Abbreviations: FDM: fused deposition model; NP: not provided; PCL: polycaprolactone; PLA: polylactic acid; PP: polypropylene; PVA: polyvinyl alcohol; SEM: scanning electron microscopy).

Device Developed	FDM Printing Parameters	Main Experimental Procedures	References
PLA filaments containing drugs: gentamicin or methotrexate (two concentrations: 1% and 2.5% *w*/*w*).	Extrusion temperature: 175 °C for gentamicin-loaded filaments; 160 °C for methotrexate-loaded filaments Speed: NP Layer height: NP	In vitro: SEM visualization. Thermal stability testing of drugs (220 °C and 190 °C for gentamicin and methotrexate, respectively). Antibacterial activity of gentamicin-loaded filaments (*Escherichia coli*): agar diffusion and liquid nutrient tests (24 h, 37 °C). Cytocompatibility of methotrexate-loaded filaments (CRL2836 osteosarcoma cells).	[94]
PLA meshes and filaments containing 1% (*w*/*w*) gentamicin.	Extrusion temperature: 175 °C Speed: NP Layer height: NP Mesh pore size: NP	In vitro: SEM visualization. Antibacterial activity (*Escherichia coli*; *Staphylococcus aureus*): agar diffusion tests (24 h, 37 °C).	[99]
PCL meshes containing gentamicin, with or without sodium alginate encapsulation (in vitro: ~10 µg; in vivo: ~1.25 mg. Deposited at six random points).	Extrusion temperature: NP Speed: 10 mm/s, flow 1 mm/s Layer height: NP Mesh pore size: two designs, macroporous (1.25 × 1.25 mm) and microporous (0.75 × 0.75 mm)	In vitro: Antibacterial activity (*Escherichia coli*): agar diffusion tests (24 h, 37 °C). In vivo: Supra-muscular implant (2 × 2 cm) in abdominal wall of female Wistar rats (n = 40, four study groups). Clean surgery. Groups (4): PCL/PCL + alginate/PCL + gentamicin/PCL + gentamicin + alginate Histopathological assessment at 7 days postimplant.	[100]
PP and PVA meshes loaded with ciprofloxacin (3 ± 1% *w*/*w* in PP; 5 ± 1% *w*/*w* in PVA).	Extrusion temperature: 190 °C for PP; 200 °C for PVA Speed: 90 mm/s for PP; 150 mm/s for PVA Layer height: 0.2 mm Mesh pore size: two designs of diameters 2 or 3 mm. 32 mesh configurations (variables: chemical composition; drug; pore size; thread width; pore shape; no. pores/100 cm^2^)	In vitro: Drug-release assay (Type V USP dissolution method). Differential scanning calorimetry. Mechanical response (tensile strength assessment). In vivo: Incisional abdominal wall defect (1 cm) repaired by the implant of meshes (1.5 × 1.5 cm) facing the abdominal cavity in rabbits (n = 20; five groups). Clean surgery. Configuration chosen for all the printed meshes: pore size 3 mm; mesh width 1.2 mm. Groups (5): PP/PVA/PP + ciprofloxacin/PVA + ciprofloxacin/Marketed PP. Macroscopic and histopathological assessment at postoperative week 6.	[101]

## Data Availability

Not applicable.

## References

[1] Brown S.H., Ward S.R., Cook M.S., Lieber R.L. (2011). Architectural analysis of human abdominal wall muscles: Implications for mechanical function. Spine.

[2] Hope W.W., Abdul W., Winters R. (2021). Abdominal Wall Reconstruction.

[3] Ger R. (2009). The clinical anatomy of the anterolateral abdominal wall musculature. Clin. Anat..

[4] Patel N.G., Ratanshi I., Buchel E.W. (2018). The best of abdominal wall reconstruction. Plast. Reconstr. Surg..

[5] Deeken C.R., Lake S.P. (2017). Mechanical properties of the abdominal wall and biomaterials utilized for hernia repair. J. Mech Behav. Biomed. Mater..

[6] Brown S.H., McGill S.M. (2010). A comparison of ultrasound and electromyography measures of force and activation to examine the mechanics of abdominal wall contraction. Clin. Biomech..

[7] Ozdogan M., Yildiz F., Gurer A., Orhun S., Kulacoglu H., Aydin R. (2006). Changes in collagen and elastic fiber contents of the skin, rectus sheath, transversalis fascia and peritoneum in primary inguinal hernia patients. Bratisl. Lek. Listy.

[8] Hernández-Gascón B., Mena A., Peña E., Pascual G., Bellón J.M., Calvo B. (2013). Understanding the passive mechanical behavior of the human abdominal wall. Ann. Biomed. Eng..

[9] Förstemann T., Trzewik J., Holste J., Batke B., Konerding M.A., Wolloscheck T., Hartung C. (2011). Forces and deformations of the abdominal wall—A mechanical and geometrical approach to the linea alba. J. Biomech..

[10] Song C., Alijani A., Frank T., Hanna G., Cuschieri A. (2006). Elasticity of the living abdominal wall in laparoscopic surgery. J. Biomech..

[11] Kalaba S., Gerhard E., Winder J.S., Pauli E.M., Haluck R.S., Yang J. (2016). Design strategies and applications of biomaterials and devices for hernia repair. Bioact. Mater..

[12] Muysoms F.E., Miserez M., Berrevoet F., Campanelli G., Champault G.G., Chelala E., Dietz U.A., Eker H.H., El Nakadi I., Hauters P. (2009). Classification of primary and incisional abdominal wall hernias. Hernia.

[13] Bellón J.M., Bajo A., García-Honduvilla N., Gimeno M.J., Pascual G., Guerrero A., Buján J. (2001). Fibroblasts from the transversalis fascia of young patients with direct inguinal hernias show constitutive MMP-2 overexpression. Ann. Surg..

[14] Franz M.G. (2006). The biology of hernias and the abdominal wall. Hernia.

[15] Pascual G., Rodríguez M., Gómez-Gil V., Trejo C., Buján J., Bellón J.M. (2010). Active matrix metalloproteinase-2 upregulation in the abdominal skin of patients with direct inguinal hernia. Eur. J. Clin. Investig..

[16] Burcharth J., Pommergaard H.C., Rosenberg J. (2013). The inheritance of groin hernia: A systematic review. Hernia.

[17] Öberg S., Andresen K., Rosenberg J. (2017). Etiology of inguinal hernias: A comprehensive review. Front. Surg..

[18] Thankam F.G., Larsen N.K., Varghese A., Bui T.N., Reilly M., Fitzgibbons R.J., Agrawal D.K. (2021). Biomarkers and heterogeneous fibroblast phenotype associated with incisional hernia. Mol. Cell Biochem..

[19] Kingsnorth A., LeBlanc K. (2003). Hernias: Inguinal and incisional. Lancet.

[20] HerniaSurge Group (2018). International guidelines for groin hernia management. Hernia.

[21] Pandey S., Sumant O. (2020). Hernia Repair Devices and Consumables Market by Product (Fixation Devices and Consumables), Surgery Type (Open Tension-Free Repair Surgery and Laparoscopic Surgery), and Hernia Type (Incisional Hernia, Umbilical, Inguinal Hernia, and Femoral Hernia): Global Opportunity Analysis and Industry Forecast, 2020–2027.

[22] Rastegarpour A., Cheung M., Vardhan M., Ibrahim M.M., Butler C.E., Levinson H. (2016). Surgical mesh for ventral incisional hernia repairs: Understanding mesh design. Plast. Surg..

[23] Luijendijk R.W., Hop W.C., van den Tol M.P., de Lange D.C., Braaksma M.M., IJzermans J.N., Boelhouwer R.U., de Vries B.C., Salu M.K., Wereldsma J.C. (2000). A comparison of suture repair with mesh repair for incisional hernia. N. Engl. J. Med..

[24] Bilsel Y., Abci I. (2012). The search for ideal hernia repair; mesh materials and types. Int. J. Surg..

[25] Bellón J.M. (2014). Classification of prosthetic materials used in hernia repair according to their structure and behavior with regard to tissue integration. Rev. Hisp. Hernia.

[26] Cevasco M., Itani K.M. (2012). Ventral hernia repair with synthetic, composite, and biologic mesh: Characteristics, indications, and infection profile. Surg. Infect..

[27] Le D., Deveney C.W., Reaven N.L., Funk S.E., McGaughey K.J., Martindale R.G. (2013). Mesh choice in ventral hernia repair: So many choices, so little time. Am. J. Surg..

[28] Franklin M.E., Gonzalez J.J., Michaelson R.P., Glass J.L., Chock D.A. (2002). Preliminary experience with new bioactive prosthetic material for repair of hernias in infected fields. Hernia.

[29] Miserez M., Jairam A.P., Boersema G.S.A., Bayon Y., Jeekel J., Lange J.F. (2019). Resorbable synthetic meshes for abdominal wall defects in preclinical setting: A literature review. J. Surg Res..

[30] Mori da Cunha M.G.M.C., Hympanova L., Rynkevic R., Mes T., Bosman A.W., Deprest J. (2019). Biomechanical behaviour and biocompatibility of ureidopyrimidinone-polycarbonate electrospun and polypropylene meshes in a hernia repair in rabbits. Materials.

[31] Sanders D.L., Kingsnorth A.N. (2012). Prosthetic mesh materials used in hernia surgery. Expert Rev. Med. Devices.

[32] Harris H.W., Primus F., Young C., Carter J.T., Lin M., Mukhtar R.A., Yeh B., Allen E.G., Freise C., Kim E. (2021). Preventing recurrence in clean and contaminated hernias using biologic versus synthetic mesh in ventral hernia repair: The PRICE randomized clinical trial. Ann. Surg..

[33] Shankaran V., Weber D.J., Reed R.L., Luchette F.A. (2011). A review of available prosthetics for ventral hernia repair. Ann. Surg..

[34] FitzGerald J.F., Kumar A.S. (2014). Biologic versus synthetic mesh reinforcement: What are the pros and cons?. Clin. Colon Rectal Surg..

[35] Patel H., Ostergard D.R., Sternschuss G. (2012). Polypropylene mesh and the host response. Int. Urogynecol. J..

[36] Totten C., Becker P., Lourd M., Roth J.S. (2019). Polyester vs polypropylene, do mesh materials matter? A meta-analysis and systematic review. Med. Devices.

[37] Alarcón I., Balla A., Soler-Frías J.R., Barranco A., Bellido-Luque J., Morales-Conde S. (2020). Polytetrafluoroethylene versus polypropylene mesh during laparoscopic totally extraperitoneal (TEP) repair of inguinal hernia: Short- and long-term results of a double-blind clinical randomized controlled trial. Hernia.

[38] Verbo A., Pafundi P., Manno A., Baccaro R., Veneziani A., Colli R., Coco C. (2016). Polyvinylidene fluoride mesh (PVDF, DynaMesh^®^-IPOM) in the laparoscopic treatment of incisional hernia: A prospective comparative trial versus Gore^®^ ePTFE DUALMESH^®^ Plus. Surg. Technol. Int..

[39] Lambertz A., van den Hil L.C.L., Schöb D.S., Binnebösel M., Kroh A., Klinge U., Neumann U.P., Klink C.D. (2016). Analysis of adhesion formation of a new elastic thermoplastic polyurethane (TPU) mesh in comparison to polypropylene (PP) meshes in IPOM position. J. Mech. Behav. Biomed. Mater..

[40] Levy A.S., Bernstein J.L., Premaratne I.D., Rohde C.H., Otterburn D.M., Morrison K.A., Lieberman M., Pomp A., Spector J.A. (2021). Poly-4-hydroxybutyrate (Phasix™) mesh onlay in complex abdominal wall repair. Surg. Endosc..

[41] Renard Y., de Mestier L., Henriques J., de Boissieu P., de Mestier P., Fingerhut A., Palot J.P., Kianmanesh R. (2020). Absorbable polyglactin vs. non-cross-linked porcine biological mesh for the surgical treatment of infected incisional hernia. J. Gastrointest. Surg..

[42] Ye X., Han X., Wei B., Huang J., Yang X., Tang X., Zheng Z., Luo L., Zhan Z., Wei H. (2018). Ventral hernia repair in rat using nanofibrous polylactic acid/polypropylene meshes. Nanomedicine.

[43] Fatkhudinov T., Tsedik L., Arutyunyan I., Lokhonina A., Makarov A., Korshunov A., Elchaninov A., Kananykhina E., Vasyukova O., Usman N. (2019). Evaluation of resorbable polydioxanone and polyglycolic acid meshes in a rat model of ventral hernia repair. J. Biomed. Mater. Res. B Appl. Biomater..

[44] Borman D.A., Sunshein K.E., Stigall K.S., Madabhushi V.V., Davenport D.L., Plymale M.A., Roth J.S. (2019). Clinical and quality of life assessment of patients undergoing laparoscopic hiatal hernia repair. Am. Surg..

[45] Sezer U.A., Sanko V., Gulmez M., Aru B., Sayman E., Aktekin A., Vardar Aker F., Demirel G.Y., Sezer S. (2019). Polypropylene composite hernia mesh with anti-adhesion layer composed of polycaprolactone and oxidized regenerated cellulose. Mater. Sci. Eng. C Mater. Biol. Appl..

[46] Shokrollahi M., Bahrami S.H., Nazarpak M.H., Solouk A. (2020). Biomimetic double-sided polypropylene mesh modified by DOPA and ofloxacin loaded carboxyethyl chitosan/polyvinyl alcohol-polycaprolactone nanofibers for potential hernia repair applications. Int. J. Biol. Macromol..

[47] Usher F.C. (1959). Further observations the use of Marlex mesh: A new technique for the repair of inguinal hernias. Am. J. Surg..

[48] Usher F.C., Cogan J.E., Lowry T. (1960). A new technique for the repair of inguinal and incisional hernias. Arch. Surg..

[49] Antonopoulos I.M., Nhas W.C., Mazzuchi E., Piovesan A.C., Birolini C., Lucon A.M. (2005). Is polypropylene mesh safe and effective for repairing infected incisional hernia in renal transplant recipients?. Urology.

[50] Alaedeen D.I., Lipman J., Medalie D., Rosen M.J. (2007). The single-staged approach to the surgical management of abdominal wall hernias in contaminated fields. Hernia.

[51] Rodríguez M., Gómez-Gil V., Pérez-Köhler B., Pascual G., Bellón J.M. (2021). Polymer hernia repair materials: Adapting to patient needs and surgical techniques. Materials.

[52] Manzini G., Henne-Bruns D., Kremer M. (2019). Severe complications after mesh migration following abdominal hernial repair: Report of two cases and review of literature. GMS Interdiscip. Plast. Reconstr. Surg. DGPW.

[53] Schmidt A., Taylor D. (2021). Erosion of soft tissue by polypropylene mesh products. J. Mech. Behav. Biomed. Mater..

[54] Nienhuijs S., Staal E., Strobbe L., Rosman C., Groenewoud H., Bleichrodt R. (2007). Chronic pain after mesh repair of inguinal hernia: A systematic review. Am. J. Surg..

[55] Bittner R., Schmedt C.G., Leibl B.J., Schwarz J. (2011). Early postoperative and one year results of a randomized controlled trial comparing the impact of extralight titanized polypropylene mesh and traditional heavyweight polypropylene mesh on pain and seroma production in laparoscopic hernia repair (TAPP). World J. Surg..

[56] Felemovicius I., Bonsack M.E., Hagerman G., Delaney J.P. (2004). Prevention of adhesions to polypropylene mesh. J. Am. Coll. Surg..

[57] Di Muria A., Formisano V., Di Carlo F., Aveta A., Giglio D. (2007). Small bowel obstruction by mesh migration after umbilical hernia repair. Ann. Ital. Chir..

[58] Basoglu M., Yildirgan M.I., Yilmaz I., Balik A., Celebi F., Atamanalp S.S., Polat K.Y., Oren D. (2004). Late complications of incisional hernias following prosthetic mesh repair. Acta Chir. Belg..

[59] Carbonell A.M., Kercher K.W., Sin R.F., Heniford B.T. (2005). Susceptibility of prosthetic biomaterials to infection. Surg. Endosc..

[60] Russo Serafini M., Medeiros Savi F., Ren J., Bas O., O’Rourke N., Maher C., Hutmacher D.W. (2021). The patenting and technological trends in hernia mesh implants. Tissue Eng. Part B Rev..

[61] Wales E., Holloway S. (2019). The use of prosthetic mesh for abdominal wall repairs: A semi-systematic-literature review. Int. Wound J..

[62] Liaw C.Y., Guvendiren M. (2017). Current and emerging applications of 3D printing in medicine. Biofabrication.

[63] Ravia T., Ranganathanb R., Pugalendhic A., Arumugamd S. (2020). 3D printed patient specific models from medical imaging—A general workflow. Mater. Today Proc..

[64] Mitsouras D., Liacouras P., Imanzadeh A., Giannopoulos A.A., Cai T., Kumamaru K.K., George E., Wake N., Caterson E.J., Pomahac B. (2015). Medical 3D printing for the radiologist. Radiographics.

[65] Liacourras P.C., Sahajwalla D., Beachler M.D., Sleeman T., Ho V.B., Lichtenberger J.P. (2017). Using computed tomography and 3D printing to construct custom prosthetics attachments and devices. 3D Print. Med..

[66] Hull C.W. (2015). The birth of 3D printing. Res. Technol. Manag..

[67] Jain A., Bansal K.K., Tiwari A., Rosling A., Rosenholm J.M. (2018). Role of polymers in 3D printing technology for drug delivery—An overview. Curr. Pharm. Des..

[68] Pravin S., Sudhir A. (2018). Integration of 3D printing with dosage forms: A new perspective for modern healthcare. Biomed. Pharm..

[69] Calignano F., Galati M., Iuliano L., Minetola P. (2019). Design of additively manufactured structures for biomedical applications: A review of the additive manufacturing processes applied to the biomedical sector. J. Healthc. Eng..

[70] Beg S., Almalki W.H., Malik A., Farhan M., Aatif M., Rahman Z., Alruwaili N.K., Alrobaian M., Tarique M., Rahman M. (2020). 3D printing for drug delivery and biomedical applications. Drug Discov. Today.

[71] Hodgdon T., Danrad R., Patel M.J., Smith S.E., Richardson M.L., Ballard D.H., Ali S., Trace A.P., DeBenedectis C.A., Zygmont M.E. (2018). Logistics of Three-dimensional Printing: Primer for Radiologists. Acad. Radiol..

[72] Pantermehl S., Emmert S., Foth A., Grabow N., Alkildani S., Bader R., Barbeck M., Jung O. (2021). 3D printing for soft tissue regeneration and applications in medicine. Biomedicines.

[73] Ventola C.L. (2014). Medical applications for 3D printing: Current and projected uses. Pharm. Ther..

[74] Ballard D.H., Trace A.P., Ali S., Hodgdon T., Zygmont M.E., DeBenedectis C.A., Smith S.E., Richardson M.L., Patel M.J., Decker S.J. (2018). Clinical applications of 3D printing: Primer for radiologists. Acad. Radiol..

[75] Dutra G.V.S., Neto W.S., Dutra J.P.S., Machado F. (2020). Implantable medical devices and tissue engineering: An overview of manufacturing processes and the use of polymeric matrices for manufacturing and coating their surfaces. Curr. Med. Chem..

[76] Jain K., Shukla R., Yadav A., Ujjwal R.R., Flora S.J.S. (2021). 3D printing in development of nanomedicines. Nanomaterials.

[77] Awad A., Fina F., Goyanes A., Gaisford S., Basit A.W. (2021). Advances in powder bed fusion 3D printing in drug delivery and healthcare. Adv. Drug Deliv. Rev..

[78] Guvendiren M., Molde J., Soares R.M., Kohn J. (2016). Designing Biomaterials for 3D Printing. ACS Biomater. Sci. Eng..

[79] Sta Agueda J.R.H., Chen Q., Maalihan R.D., Ren J., da Silva I.G.M., Dugos N.P., Caldona E.B., Advincula R.C. (2021). 3D printing of biomedically relevant polymer materials and biocompatibility. MRS Commun..

[80] Casanellas I., García-Lizarribar A., Lagunas A., Samitier J. (2018). Producing 3D biomimetic nanomaterials for musculoskeletal system regeneration. Front. Bioeng. Biotechnol..

[81] Derakhshanfar S., Mbeleck R., Xu K., Zhang X., Zhong W., Xing M. (2018). 3D bioprinting for biomedical devices and tissue engineering: A review of recent trends and advances. Bioact. Mater..

[82] Zhang J., Wehrle E., Rubert M., Müller R. (2021). 3D Bioprinting of human tissues: Biofabrication, bioinks, and bioreactors. Int. J. Mol. Sci..

[83] Di Prima M., Coburn J., Hwang D., Kelly J., Khairuzzaman A., Ricles L. (2016). Additively manufactured medical products—The FDA perspective. 3D Print. Med..

[84] Mirza M.A., Iqbal Z. (2018). 3D printing in pharmaceuticals: Regulatory perspective. Curr. Pharm. Des..

[85] Eisenstein M. (2015). First 3D-printed pill. Nat. Biotechnol..

[86] Ballard D.H., Jammalamadaka U., Tappa K., Weisman J.A., Boyer C.J., Alexander J.S., Woodard P.K. (2018). 3D printing of surgical hernia meshes impregnated with contrast agents: In vitro proof of concept with imaging characteristics on computed tomography. 3D Print. Med..

[87] Yang Z., Song Z., Nie X., Guo K., Gu Y. (2020). A smart scaffold composed of three-dimensional printing and electrospinning techniques and its application in rat abdominal wall defects. Stem. Cell Res..

[88] Pensa N.W., Curry A.S., Bonvallet P.P., Bellis N.F., Rettig K.M., Reddy M.S., Eberhardt A.W., Bellis S.L. (2019). 3D printed mesh reinforcements enhance the mechanical properties of electrospun scaffolds. Biomater. Res..

[89] Shin C.S., Cabrera F.J., Lee R., Kim J., Veettil R.A., Zaheer M., Adumbumkulath A., Mhatre K., Ajayan P.M., Curley S.A. (2021). 3D-bioprinted inflammation modulating polymer scaffolds for soft tissue repair. Adv. Mater..

[90] Chung J.J., Im H., Kim S.H., Park J.W., Jung Y. (2020). Toward biomimetic scaffolds for tissue engineering: 3D printing techniques in regenerative medicine. Front. Bioeng. Biotechnol..

[91] Mir M., Ansari U., Najabat Ali M. (2017). Macro-scale model study of a tunable drug dispensation mechanism for controlled drug delivery in potential wound-healing applications. J. Appl. Biomater. Funct. Mater..

[92] Do A.V., Worthington K.S., Tucker B.A., Salem A.K. (2018). Controlled drug delivery from 3D printed two-photon polymerized poly(ethylene glycol) dimethacrylate devices. Int. J. Pharm..

[93] Ballard D.H., Tappa K., Boyer C.J., Jammalamadaka U., Hemmanur K., Weisman J.A., Alexander J.S., Mills D.K., Woodard P.K. (2019). Antibiotics in 3D-printed implants, instruments and materials: Benefits, challenges and future directions. J. 3D Print. Med..

[94] Weisman J.A., Nicholson J.C., Tappa K., Jammalamadaka U., Wilson C.G., Mills D.K. (2015). Antibiotic and chemotherapeutic enhanced three-dimensional printer filaments and constructs for biomedical applications. Int. J. Nanomed..

[95] Huang Y., Zhang X.F., Gao G., Yonezawa T., Cui X. (2017). 3D bioprinting and the current applications in tissue engineering. Biotechnol. J..

[96] Tappa K., Jammalamadaka U., Ballard D.H., Bruno T., Israel M.R., Vemula H., Meacham J.M., Mills D.K., Woodard P.K., Weisman J.A. (2017). Medication eluting devices for the field of OBGYN (MEDOBGYN): 3D printed biodegradable hormone eluting constructs, a proof of concept study. PLoS ONE.

[97] Farmer Z.L., Utomo E., Domínguez-Robles J., Mancinelli C., Mathew E., Larrañeta E., Lamprou D.A. (2021). 3D printed estradiol-eluting urogynecological mesh implants: Influence of material and mesh geometry on their mechanical properties. Int. J. Pharm..

[98] Koons G.L., Mikos A.G. (2019). Progress in three-dimensional printing with growth factors. J. Control. Release.

[99] Ballard D.H., Weisman J.A., Jammalamadaka U., Tappa K., Alexander J.S., Griffen F.D. (2017). Three-dimensional printing of bioactive hernia meshes: In vitro proof of principle. Surgery.

[100] Calero Castro F.J., Yuste Y., Pereira S., Garvín M.D., López García M.A., Padillo F.J., de la Portilla F. (2019). Proof of concept, design, and manufacture via 3-D printing of a mesh with bactericidal capacity: Behaviour in vitro and in vivo. J. Tissue Eng. Regen. Med..

[101] Qamar N., Abbas N., Irfan M., Hussain A., Arshad M.S., Latif S., Mehmood F., Ghori M.U. (2019). Personalized 3D printed ciprofloxacin impregnated meshes for the management of hernia. J. Drug Deliv. Sci. Technol..

[102] Bose S., Robertson S.F., Bandyopadhyay A. (2018). Surface modification of biomaterials and biomedical devices using additive manufacturing. Acta Biomater..

[103] Durfee W.K., Iaizzo P.A., Iaizzo P.A. (2019). Medical applications of 3D printing. Engineering in Medicine. Advances and Challenges.

[104] Anurov M.V., Titkova S.M., Oettinger A.P. (2012). Biomechanical compatibility of surgical mesh and fascia being reinforced: Dependence of experimental hernia defect repair results on anisotropic surgical mesh positioning. Hernia.

[105] Astruc L., De Meulaere M., Witz J.F., Novácek V., Turquier F., Hoc T., Brieu M. (2018). Characterization of the anisotropic mechanical behavior of human abdominal wall connective tissues. J. Mech. Behav. Biomed. Mater..

[106] Hernández-Gascón B., Peña E., Pascual G., Rodríguez M., Bellón J.M., Calvo B. (2012). Long-term anisotropic mechanical response of surgical meshes used to repair abdominal wall defects. J. Mech. Behav. Biomed. Mater..

[107] Lambertz A., Vogels R.R., Binnebösel M., Schöb D.S., Kossel K., Klinge U., Neumann U.P., Klink C.D. (2015). Elastic mesh with thermoplastic polyurethane filaments preserves effective porosity of textile implants. J. Biomed. Mater. Res. Part A.

[108] Pott P.P., Schwarz M.L., Gundling R., Nowak K., Hohenberger P., Roessner E.D. (2012). Mechanical properties of mesh materials used for hernia repair and soft tissue augmentation. PLoS ONE.

[109] Hernández-Gascón B., Espés N., Peña E., Pascual G., Bellón J.M., Calvo B. (2014). Computational framework to model and design surgical meshes for hernia repair. Comput. Methods Biomech. Biomed. Eng..

[110] Cobb W.S., Peindl R.M., Zerey M., Carbonell A.M., Heniford B.T. (2009). Mesh terminology 101. Hernia.

[111] Cobb W.S., Burns J.M., Peindl R.D., Carbonell A.M., Matthews B.D., Kercher K.W., Heniford B.T. (2006). Textile analysis of heavy weight, mid-weight, and light weight polypropylene mesh in a porcine ventral hernia model. J. Surg. Res..

[112] Bellón J.M., Moreno-Egea A. (2021). Clinical guide for the use of prosthetic material in the repair of incisional hernia. Rev. Hisp. Hernia.

[113] Bellón J.M., Rodríguez M., García-Honduvilla N., Gómez-Gil V., Pascual G., Buján J. (2009). Comparing the behavior of different polypropylene meshes (heavy and lightweight) in an experimental model of ventral hernia repair. J. Biomed. Mater. Res. Part B Appl. Biomater..

[114] Tanikella N.G., Wittbrodt B., Pearce J.M. (2017). Tensile strength of commercial polymer materials for fused filament fabrication 3-D printing. Addit. Manuf..

[115] Mellert L.T., Cheung M.E., Zografakis J.G., Dan A.G. (2019). Laparoscopic inguinal hernia repair using progrip self-fixating mesh: Technical learning curve and mid-term outcomes. Surg. Technol. Int..

[116] Thölix A.M., Kössi J., Remes V., Scheinin T., Harju J. (2018). Lower incidence of postoperative pain after open inguinal hernia surgery with the usage of synthetic glue-coated mesh (Adhesix^®^). Am. Surg..

[117] Turza K.C., Butler C.E. (2012). Adhesions and meshes: Synthetic versus bioprosthetic. Plast. Reconstr. Surg..

[118] Choi Y.J., Kim T.G., Jeong J., Yi H.G., Park J.W., Hwang W., Cho D.W. (2016). 3D cell printing of functional skeletal muscle constructs using skeletal muscle-derived bioink. Adv. Healthc. Mater..

[119] Lui Y.S., Sow W.T., Tan L.P., Wu Y., Lai Y., Li H. (2019). 4D printing and stimuli-responsive materials in biomedical aspects. Acta Biomater..

